# Implementation of an Occupational Sun Safety Intervention: A Comparison of Two Scalability Strategies

**DOI:** 10.1097/JOM.0000000000003248

**Published:** 2024-10-10

**Authors:** David B. Buller, Mary Klein Buller, Richard Meenan, Gary R. Cutter, Julia Berteletti, Kimberly L. Henry, Alishia Kinsey, Irene Adjei, Noah Chirico

**Affiliations:** Klein Buendel, Inc., Golden, Colorado; Klein Buendel, Inc., Golden, Colorado; Kaiser Permanente Center for Health Research, Portland, Oregon; University of Alabama, Birmingham, Alabama; Klein Buendel, Inc., Golden, Colorado; Colorado State University in Ft Collins, Colorado; Klein Buendel, Inc., Golden, Colorado; Klein Buendel, Inc., Golden, Colorado; Klein Buendel, Inc., Golden, Colorado

**Keywords:** cancer, skin neoplasms, prevention, occupational, implementation

## Abstract

**Objective:**

Two methods for scaling up an evidence-based occupational sun protection program were compared.

**Methods:**

Regional districts (*n* = 138) in 21 state Departments of Transportation throughout the United States were randomized to receive the Go Sun Smart at Work program via in-person or digital scalability methods in 2019–2022 in 1:2 ratio. Managers completed pretest and posttest surveys and employees completed posttest surveys. Because of the COVID pandemic, only posttest measures were analyzed (*n* = 255 managers; *n* = 1387 employees).

**Results:**

After scale-up, more employees reported training (b = 0.381, *P* = 0.021) and communication (b = 0.112, *P* = 0.049) at workplaces in in-person rather than digital strategy. There were no differences in managers’ reports of sun protection training, communication, or actions by scalability method (*P* > 0.05).

**Conclusions:**

Occupational sun protection was implemented during program scale up and employees recalled training/communication more in the in-person than digital strategy.

In the United States, the National Institute of Environmental Health Sciences^1^ has identified ultraviolet radiation (UV) as a carcinogen, similar to asbestos, radon, benzene, and vinyl chloride. In 2019, there were an estimated 2.8 million (95% uncertainty interval [UI]: 2.5–3.2 million) new cases of basal cell skin cancer and 1.5 million (95% UI: 1.3–1.7 million) new cases of squamous cell skin cancer (ie, keratinocyte cancers of the skin), along with 82,054 (95% UI: 62,155–108,409) new cases of melanoma, in the United States.^2^ There was also an estimated 4472 (95% UI: 3934–4744) deaths from squamous cell skin cancers and 11,906 (95% UI: 8557–15,096) deaths from melanoma that year.^2^

Individuals who labor outdoors are at elevated risk for keratinocyte cancers of the skin and melanoma. Latest estimates from the World Health Organization showed a pooled relative risk (RR) of 1.60 (95% confidence interval [CI]: 1.21–2.11) for nonmelanoma skin cancers (ie, keratinocyte cancers) associated with occupational exposure to UV (1.78 [95% CI: 1.36–2.35] in studies conducted in the Americas),^3^ both squamous cell (RR = 2.43, 95% CI: 1.64–3.62) and basal cell (RR = 1.50, 95% CI: 1.10–2.04) carcinomas. Occupational UV exposure of two or more hours daily from March/April to October over many years appeared to increase the risk of keratinocyte cancer of the skin in light-skinned workers.^4^ For melanoma, RR with occupational exposure to UV was elevated only when including studies with subtype Lentigo Maligna (RR = 1.45, 95% CI: 1.08–1.94) compared to studies excluding this subtype (RR = 0.69, 95% CI: 0.55–0.86).^3^

The burden of skin cancer in death, disability, and cost is also substantial. Globally, occupational exposure to UV has been estimated to increase the risk of death from keratinocyte cancers of the skin (RR = 1.30, 95% CI: 1.14–1.47), but not from melanoma (RR = 0.99, 95% CI: 0.87–1.12).^5^ For the United States, estimated disability adjusted life years from keratinocyte cancers of the skin and from melanoma associated with occupational exposure to UV were 35.61 (95% UI: 29.29, 41.93) and 1064 (95% UI: 889, 1239), respectively.^5^ Costs associated with treatment of all skin cancers in the United States were estimated at US$8.9 billion in 2016–2018 (US$6.5 billion for keratinocyte cancers and US$2.5 billion for melanoma). ^6–8^

Because of its high prevalence and burden, the US Surgeon General^6^ issued a national call to action to reduce skin cancer and identified workplaces as a key venue for intervention to protect the estimated 18.9% (95% uncertainty range [UR]: 17.53–20.28) of the US workforce occupationally exposed to UV.^5^ Health authorities world-wide advise individuals to use shade, protective clothing, and broad-spectrum (UVA and UVB) sunscreens,^9^ but many outdoor workers do not consistently practice these personal protection behaviors.^10–15^ It has been estimated that a 10% reduction in severe sunburns would prevent 8.5% of melanomas attributable to sunburning, and regular sunscreen use would prevent 9.3% of squamous cell skin cancer and 14% of melanomas.^16^ However, protection of workers from chronic solar UV exposure has received limited attention.^17–20^

Ideal workplace interventions aimed at preventing chronic disease (ie, skin cancer) as well as acute injury (ie, sunburn)^21^ incorporate institutional preventive actions (eg, risk assessment) and policies geared to broad actions at all organizational levels (ie, environmental and administrative controls and personal protective equipment [PPE]), along with employee education, to motivate and support personal sun protection practices by conveying perceptions of risk, knowledge, and skills for effectively using PPE, and creating workplace norms for sun protection.^22–29^ Policy and education can work synergistically to integrate health promotion and safety procedures,^30^ clarify personal and organizational responsibilities, and formally direct employees to take precautions,^31,32^ which can overcome low perceived risk^33^ and personal preferences,^34^ equalize gender and age differences,^35–37^ and address organizational and setting barriers.^38,39^ However, regulations aimed at reducing occupational UV exposure are limited in most counties except Australia and New Zealand.^28^ They also can increase managers’ support for sun safety that has been associated with improved employee sun protection.^32^ Multilevel sun safety interventions have been effective in communities^40^ and sun protection interventions in occupational settings have been effective,^41–43^ including interventions promoting institutional changes (risk assessments and policy change) and delivering education and communication on sun safety to employees. In a series of randomized trials, the authors created an evidence-based occupational skin cancer prevention program^44–46^ that increased the number of employers adopting sun safety policies and implementing sun safety training and other sun protective actions, and improved employees’ sun protection behaviors.^47–49^

To realize the promise of effective prevention interventions, scalability strategies should be evaluated to ensure wide, equitable, cost-effective reach, but research on optimal scalability strategies is limited.^50^ Scalability is the “ability of a health intervention shown to be efficacious on a small scale and/or under controlled conditions to be expanded under real world conditions to reach a greater proportion of the eligible population while retaining effectiveness.”^50^ It is related to fit of interventions with local settings and organizational infrastructure and to cost of distribution.^51^ However, at scale, intervention effectiveness can decline because intervention fidelity often decreases and adaptations to fit organizational parameters (ie, budget, procedures, and context) are needed during scale-up.^50,52,53^

This study was designed to compare two methods for scaling up the authors’ occupational sun protection intervention in the nationwide public-sector transportation industry (ie, state Departments of Transportation^54^ [DOTs]). Scalability was guided by the RE-AIM framework. RE-AIM has been applied to integrating policies with health promotion,^55^ as this evidence-based intervention does. An in-person dissemination method with face-to-face on-site visits to the workplaces was compared to a digital dissemination method that utilized online platforms and resources to replace face-to-face on-site contact. In both methods, intervention implementation was tailored to employer’s readiness to implement skin cancer prevention based on diffusion of innovations theory.^56^ In this paper, secondary analyses are reported that examined differences between dissemination methods in program implementation and employee and manager sun protection practices. The primary analysis comparing cost-effectiveness of scalability methods will be reported elsewhere. It was hypothesized that program implementation would be greater with the in-person than digital scalability method.

## METHODS

The experimental design and scalability procedures were described in detail previously^54^ and are summarized here. The COVID pandemic responses by the state governments interrupted and limited the scalability procedures, as we detail below.

### Sample and Recruitment Procedures

With the cooperation of the American Association of State Highway and Transportation Officials (AASHTO), the 50 US state DOTs were invited to participate by a letter and description sent from the AASHTO Chief Executive Officer and follow-up telephone and email contact by project staff. DOTs are state agencies charged with planning, building, operating, maintaining, and regulating the transportation infrastructure (eg, roadways, railways, airports, waterways, ports, bridges, and mass transportation) within state boundaries, using state and federal funds. Twenty-one state DOTs from all four US Census regions agreed to participate. Regional districts (*n* = 138) within each participating DOT were the units of randomization. To be eligible, districts a) were in a participating state DOT, b) were located in the United States, c) had the state DOT provide written safety policies to project staff, and d) had managers complete the pretest survey. A priori power calculations showed that with approximately 90 districts assigned to the digital method and 36 assigned to the in-person method, there was at least 80% power to detect a difference in proportion implementing training of 0.060 versus 0.300 in each group, respectively. Management of each participating DOT designated a key contact manager at the state office. Eligibility criteria for district managers were a) being in a management or front-line supervisory position (ie, safety officers/leads, safety coordinators, maintenance administrators/supervisors, resident/construction engineers, maintenance/district superintendents, operations/infrastructure managers, and project/section supervisors); b) supervising outdoor workers; c) consenting to participate, and d) completing the pretest. Managers were sampled by position at pretest, and we attempted to posttest the same persons holding those positions. If the position holder changed, we tried to assess the new position holder, but most DOT managers were unwilling to provide email addresses to follow managers who departed. Managers were invited to complete the pretest and posttest survey online (with weekly reminders); persistent nonresponders received a printed survey by US mail. Employees in the regional districts were invited to complete a posttest. To be eligible, employees had to a) be employed part/full-time, b) work at least part of daytime hours outdoors, and c) provide consent. Typical outdoor jobs included road, bridge, and traffic crews, construction crews, maintenance crews, machine operators, motor patrols, drivers, and engineers. Road construction, maintenance, trades, and transportation occupations have been associated with high UV exposure in summer months in temperate regions.^57–59^ To maintain anonymity, employee survey links were sent to district contacts to distribute to employee listservs. Paper surveys were also mailed to district locations to distribute in public spaces.

### Experimental Design

The managers and employees of regional districts in the participating DOTs were enrolled in a randomized, pretest-posttest two-group experimental design. The project statistician randomized districts into one of two scalability strategies, after pretesting, in a 1:2 ratio. The two groups differed in the method for distributing and supporting implementation of Go Sun Smart at Work (GSS@W). One-third of districts (*n* = 46) were assigned to the in-person method (IP), in which research staff met face-to-face on-site with district managers and delivered training to employees. This method was originally used in the effectiveness trials on the occupational sun protection intervention.^60^ Printed materials were distributed by mail to the regional districts. The remaining districts (*n* = 92) were assigned to a digital method, in which research staff used email and telephone communication for virtual meetings with district contacts and an online video to train employees was done via online video. Video training and printed materials were available on an intervention website or provided as separate files, as requested. In both conditions, research staff followed up with managers by telephone and email. The intervention period occurred in 2019 to 2022. Posttesting occurred 22 to 40 months after randomization. All procedures were approved by the WCG Institutional Review Board.

### Changes to the Design

Changes were made to the design initially to increase its feasibility and then later to cope with disruption created by states’ COVID pandemic responses. The sample was adjusted to 138 regional DOT districts because of budget constraints from the awarding agency, and allocation ratio to scalability strategy was adjusted to 1:2 (in-person to digital) to maintain statistical power. It also included just state DOTs to leverage the strong support from AASHTO for recruiting DOTs and similar organizational structure of DOTs, with regional districts that could be randomized. However, this necessitated altering the policy measure to be a moderator rather than an outcome, because policies were adopted at the state, not regional, levels. In addition, with randomization of districts occurring within states, we did not stratify the districts a priori on size or solar intensity. Posttesting was originally scheduled for 20 months following randomization but was delayed in some DOTs by the pandemic disruption. Employees were only posttested to reduce project costs and overcome state DOT managers’ reluctance to provide access to them initially.

The states’ responses to the COVID pandemic limited our access to the regional districts in 2020–2022. Prior to 2020, 116 DOT districts had been randomized and project staff were using the scalability strategies to disseminate the GSS@W program to 101 of these workplaces. During the pandemic, many DOT employees were classified as essential and continued to work on-site, but nearly all participating DOTs eliminated face-to-face on-site contact and meetings both with representatives of outside organizations and among the employees themselves. Thus, we were unable to conduct in-person meetings and trainings. In addition, many of the educational activities at the workplaces in both the in-person and digital scalability conditions were substantially reduced in scope, with COVID prevention taking priority over other health and safety issues. In 2021 and 2022, states relaxed some of these restrictions and we were able to restart the on-site meetings and trainings in a few states. Furthermore, the state DOT managers reported that the DOTs experienced substantial manager and employee turnover due to retirements and health impacts of COVID. Thus, DOTs’ commitment to the project declined and many managers and employees who we tried to assess were unavailable or unresponsive at posttest. Thus, we included all managers and employees who completed the posttest, regardless of time spent working outdoors, and controlled for time spent working outdoors in the analyses. We also included a measure of changes in the workplaces due to the COVID response that might have affected the implementation of GSS@W (described below) and treated it as a covariate.

### Go Sun Smart at Work Intervention

**FIGURE 1.**
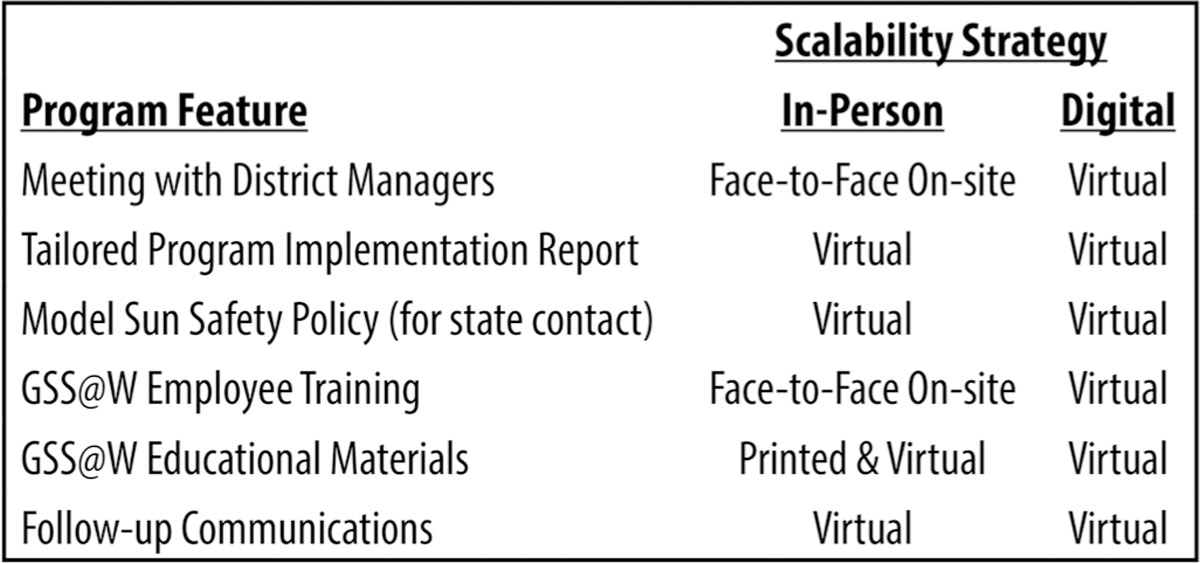
Comparison of features of in-person and digital scalability strategies.

The GSS@W program was described in detail in previous publications^47–49^ and is briefly described here. GSS@W aimed to increase occupational sun protection by promoting the adoption of sun protection policies by employers and personal sun protection behaviors by employees. It was based primarily on principles of diffusion of innovations theory^56^ and theories of relationship development.^61–65^ Policy adoption and implementation of training and communication to employees on personal protection were motivated by increasing perceived need for workplace sun safety among managers and showing them that workplace policy and employee education fit the organization and was consistent with advice from national health authorities. An intervention staff person was assigned to each regional district and served as a Sun Safety Coordinator throughout the intervention period. In meetings and follow-up communication with managers, the Sun Safety Coordinator aimed to obtain management commitment to occupational sun safety^66–68^ and help managers to plan for policy implementation, make changes in the program or the organization to improve its fit, and communicate about changes to promote sun safety to affected managers and employees.^69–71^ The key components of GSS@W were meetings with managers, employee training, materials promoting sun protection policies and education, and monthly follow-up contacts. These components promoted personal sun safety practices at the workplace and reinforced and supported implementation and maintenance of policy, training, and other workplace communication on sun safety over time. The 30-minute employee training consisted of sections on a) The US Skin Cancer Problem; b) The Sun, UV Rays and Skin Cancer; c) Assessing Your Personal Risk; and d) Practicing Sun Safety. The GSS@W website had a collection of materials to support policy development and implementation and educate employees and promote sun safety practices (ie, worksite audit and facts sheets on sun safety and barriers to policy implementation and theory-based posters, risk assessment brochures, and tip cards for employees) (see examples in Supplemental Content Figure 1, http://links.lww.com/JOM/B733).

### Scalability Strategies

The GSS@W program was delivered to the regional districts by an in-person or digital scalability strategy that differed in the mode of the initial meetings with managers and presentation of the GSS@W training to employees. Figure 1 presents a comparison of the features of the scalability strategies.

### In-Person Strategy

The in-person scalability strategy was the method used to implement GSS@W in the previous randomized trial testing intervention effectiveness.^60^ The meetings with senior managers and training of employees were conducted face-to-face on-site at the DOT regional districts by the Sun Safety Coordinator. In most cases, a single key contact manager met with the Coordinator, but the Sun Safety Coordinator tried to meet with other managers to increase knowledge sharing, idea generation, synergistic conversations, and collaborative learning.^72,73^ Given the size of the regional districts, on-site meetings with senior managers usually occurred at the district’s main office, but on-site trainings were presented at multiple times at different maintenance garages throughout the region. Training usually occurred at the beginning or end of the day. Sometimes, the state DOT contact manager or regional contact manager would accompany the Sun Safety Coordinator to these locations. The GSS@W website was provided to regional district managers, who were encouraged to use the promotional materials in the district. In addition, three mailings with copies of printed posters and tip cards were sent to regional managers to distribute and post in the workplaces. A model sun protection policy was provided to state-level managers by email. The Sun Safety Coordinator contacted regional managers monthly after the in-person meeting by email and telephone to maintain the relationship with managers, assist them further with training and other communications on sun protection with employees, and troubleshoot barriers to program implementation.

### Digital Strategy

The digital scalability strategy virtualized the face-to-face on-site contacts, trainings, and policy promotion, using conferencing technology, online training platform, and the GSS@W website. It had the same intervention goals, components, and schedule as the in-person method. The virtual meetings and follow-up communication between the Sun Safety Coordinator and DOT regional managers were conducted by multimodel synchronous web-based video conferencing (ie, Zoom) or telephone conference call technology. An online video version of the sun safety training was created; managers were encouraged to use it with employees in groups or individually. In some cases, files with the training were provided that could be delivered through the DOT’s learning management system. All printed materials, posters, risk assessment brochures, and tip cards were available in digital format on the GSS@W website, where managers could download them for use with employees. A model sun protection policy was provided to state-level managers by email. We explored using social media to communicate with employees in the workplace, but state DOTs’ highly restrictive cybersecurity policies limited the use of this channel.

### Tailoring Intervention Delivery on Readiness to Implement

In both scalability methods, communication by Sun Safety Coordinators with managers about implementation of GSS@W was tailored to their readiness to implement occupational sun safety based on stages in the diffusion of innovations theory: agenda setting (ie, need for sun safety), matching (ie, fit of sun safety with existing policy/procedures), structuring (ie, initial implementation of policies/actions), and clarifying (ie, communicating with employees, other managers, and clients to garner support and counter opposition). In addition, intervention staff prepared a tailored report on the DOT’s current support and actions for occupational sun safety after the first meetings with state-level senior managers. Readiness was identified from a review of existing DOT policies and safety manuals on sun safety, state and regional managers’ pretest survey responses, and a checklist of workplace sun safety actions that were already being implemented, might be implemented, or would never be implemented in the DOT, completed by managers. The report provided recommendations and a plan for implementing sun safety for employees that matched readiness. This tailoring was intended to increase the relevance of coaching provided by the Sun Safety Coordinator and the GSS@W resources selected for implementation.

### Measures

#### GSS@W Implementation

GSS@W implementation was measured in posttest surveys of managers and employees, using scales modified from our previous research.^74,75^ Managers reported whether each of the following actions occurred at the workplace (yes, no, or don’t know) since the pretest assessment:

1. Training on the health risks of sun exposure is provided to employees (including managers and supervisors).
2. Employer monitors UV Index and work schedule is adjusted for harm associated with UV level.
3. Employees are encouraged to wear UV-protective clothing or uniforms (shirts with sleeves; long pants), hats, and/or eyewear when outdoors.
4. Employees are encouraged to wear sunscreen with SPF 30 or greater when outdoors.
5. Messages are communicated to employees about protecting their skin and eyes from the sun while outdoors at work.
6. Employer provides sun protection resources, such as sunscreen, UV-protective clothing/uniforms, hats, and/or eyewear to employees. “Provides” means your employer gives employees these items or gives employees money to purchase them for use at work.
7. Temporary or permanent shade structures are provided in the work environment.
8. Employer requests that staff employed by contractors/subcontractors comply with the sun safety policy while working in my employer’s work environments.
9. Employer encourages employees to regularly check their skin for signs of skin cancer either by themselves or by a physician.
10. Employer conducts a risk assessment of sun exposure and sun protection for employees in the work environment.

Two summed implementation scores were created—training and communication implementation and sun protection actions implementation. Training and communication combined four items: employees are encouraged to wear UV-protective clothing, hats, and eyewear and to wear sunscreen, messages are communicated to employees about protecting their skin and eyes from the sun, and training on the health risks of sun exposure is provided to employees (possible range = 0–4). Sun protection actions combined all other actions listed above (possible range = 0–6). In addition, managers rated the innovation characteristics from diffusion of innovations theory of implementing sun protection at the workplace (ie, changing formal written policies, policies, and training to protect employees’ skin and eyes from the sun)—necessary, too expensive, compatible with work procedures, too complicated, acceptable to employees, and would improve that organization’s existing risk management or employee wellness program (combined score range = 1–5).

Employees reported whether they received the sun safety training, favorability toward training (5 Likert-type items on was easy to view, goals of the training were apparent, received enough information, liked the training, and was comparable to other trainings on the job; range = 1–5), and whether employers communicated about sun protection through oral, written, or electronic messages (yes, no, don’t know).^60^

### Sun Protection at Work

Sun protection behaviors at work were assessed in surveys of employees and managers. They rated the frequency of nine sun protection behaviors (1 = never, 5 = always): sunscreen with SPF 15 or greater and with SPF 30 or greater, UV-protective clothing such as long-sleeved shirts and long pants, hat with any type of brim, hat with a wide-brim all the way around (including hat with flap on the back that protects ears and neck), sunglasses, shade use, limiting exposure to midday sun, and have sunscreen, hat, and eye protection at work at all times. They also indicated whether they had been sunburned in the past year anywhere and on the job (yes/no; number of times). These are standard, validated, and reliable measures from past studies,^76–86^ which we used in the trial evaluating GSS@W effectiveness.^49^

### Manager Awareness of Occupational Sun Protection Policy

Managers reported if the DOT had a formal written policy, administrative procedure, or training standard on sun protection of employees and unwritten informal standard operating procedures on sun safety (ie, presence and content of procedures).

### Measures of Potential Effect Moderators

Potential moderators were collected in manager and employee surveys. These included number of hours spent working outdoors in a typical week in the summer (April–October) and winter (November–March), awareness of DOT policy or unwritten operating procedure on sun protection of employees, skin type (always burn and unable to tan; usually burns but can tan if work at it; sometimes mildly burns and then tans easily; rarely burns and tans easily),^87^ personal history of skin cancer, age, and race/ethnicity. Finally, managers reported on eight restrictions in the DOT workplaces in response to the 2019 novel coronavirus (COVID-19) pandemic in the posttest surveys, which was summed into a count of total restrictions (Table 1).

### Statistical Analysis Methods

Models testing treatment effects were fit with Mplus, Version 8.6 (Muthén & Muthén, Los Angeles, CA), using negative binomial regression, linear regression, and logistic regression. District was specified as the clustering variable to account for nonindependence of observations. To examine overall treatment effects, each outcome assessed at posttest was regressed on the treatment indicator (1 = in-person strategy vs 0 = digital strategy), wave (a factor with three levels—prepandemic vs early pandemic, vs late pandemic), and treatment by wave interaction. States were classified into three waves for analysis, to reflect adjustments to scalability strategies due to COVID-19 restraints: Wave 1 (pre-COVID) districts had most scalability strategy activities completed prior to the pandemic restrictions, Wave 2 (early COVID) districts started the scalability strategies prior to the pandemic but did not receive all activities (ie, some missed on-site visits for meetings with managers and employee training), and Wave 3 (late COVID) districts initiated the scalability strategies after the pandemic started and received a revised version of the strategies (ie, Zoom trainings led by Sun Safety Coordinators were offered when on-site visits were restricted). Missing data were handled by imputation using the mice package for R (R Core Team, Vienna, Austria).^88^ Rubin’s rules were used to combine estimates across imputations.^89^ Selected covariates were entered as control variables, including hours worked outside, age, skin type (1 = high risk for melanoma, 4 = low risk for melanoma), sex (male vs female), and race/ethnicity (non-Hispanic White vs other). Given the large number of respondents from the Arkansas DOT, a control variable was included comparing Arkansas to all other states. Additionally, for the outcomes reported by managers, the following control variables were included: a sum score of COVID-19 effects on the organization, pretest measure of hours worked outside, written and unwritten sun protection policies in the workplace, and a set of demographic variables of the manager (age, skin type, history of skin cancer, sex, and race/ethnicity). For outcomes reported by employees, the following control variables were included: hours the employee worked outside and a set of demographic variables of employees (age, skin type, history of cancer, sex, and race/ethnicity). Given the large loss to follow-up, posttest outcome measures were not adjusted for pretest values. We assumed that randomization equalized the in-person and digital scalability groups on pretest demographic, job, and workplace characteristics and sun protection by managers and employees in this randomized posttest-only analysis.^90^ A two-tailed *P* value of 0.05 was used in all analyses.

## RESULTS

### Demographic Profile and Job Characteristics of Manager and Employee Samples

A total of 255 managers and 1387 employees completed the posttest survey from 94 DOT districts (*n* = 34 districts, 89 managers [2.6 per district], and 534 employees [15.7 per district] in in-person strategy; *n* = 60 districts, 166 managers [2.8 per district], and 950 employees [15.8 per district] in digital strategy) (see Consolidated Standards of Reporting Trials diagram in Supplemental Digital Figure 2, http://links.lww.com/JOM/B734). Table 1 provides the demographic profile and job characteristics of managers and employees. A majority of posttested managers and employees were non-Hispanic White, about 1 in 4 managers (26.2%) and employees (27.3%) reported having sun-sensitive skin types (ie, always burn or usually), and about 1 in 10 had been diagnosed with skin cancer (12.7% of managers and 7.8% of employees). Managers and employees were predominately male (>85%), with an average age of 51.3 years for managers and 45.4 years for employees. Managers and employees spent a substantial amount of time working outdoors—17.4 hours for managers and 23.4 hours for employees on average. Managers reported some ability to make changes to workplace procedures to improve safety of employees (mean = 3.54 out of 5). As expected, DOTs made several changes to workplace procedures in response to the COVID pandemic (mean = 4.10 out of 8), the most frequent being prohibiting employees from gathering, reducing the number of employees who worked together, providing individual training online rather than in groups in-person, and closing indoor areas where employees gathered.

### Go Sun Smart at Work Program Implementation Managers

Nearly all managers (89.0%) reported implementing some form of communication and training on sun safety for employees since completing the pretest survey (ie, before the scalability strategies), with a majority (65.9%) reporting that they implemented nearly all training and communication actions assessed (ie, at least 3 out of 4 actions) (Table 2). Most managers (80.0%) also reported implementing some sun protection actions for employees during this approximately 2-year scale-up period. However, very few implemented nearly all of these actions (10.6% implemented at least 4 out of 5 actions), with a majority implementing 2 or fewer actions (55.3%) (Table 2). A majority of managers (62.0%) said that at posttest, there was a budget for employee sun protection actions at their workplaces. Less than 2 in 5 managers reported that the DOT had a written policy (27.3%) or informal standard operating procedures (39.1%) on sun protection of employees when posttested (Table 2).

### Employees

At posttest, nearly half of employees (48.9%) reported affirmatively that they received training for sun protection at the workplace (ie, on protecting their skin and eyes from the sun) (Table 2). They also reported receiving sun protection information in 1.53 out of 3 channels in the workplace (ie, written, oral, or electronic communication), on average. Employees were generally favorable toward the occupational sun protection training (mean = 3.65). Employees reported that they purchased only a few items to help them practice sun safety (ie, sunscreen with SPF 30 or higher, wide-brimmed hat, brim extenders, or flaps, sunglasses, or long-sleeved work shirts; mean = 0.70 out of 2).

### Differences in Go Sun Smart at Work Program Implementation by Scalability Strategy

There were no differences in managers’ reports of GSS@W implementation by scalability strategy or in their favorable perceptions of innovation characteristics of implementation at posttest. Neither the main effects of treatment (Tables 3, 4) nor the interaction of treatment by wave (Supplemental Digital Content Table 1, http://links.lww.com/JOM/B735) were statistically significant. However, trends in mean implementation measures in regional districts to which GSS@W was disseminated before the pandemic (Wave 1) were in the direction of more sun safety actions in the in-person than digital scalability strategy (Table 3), and managers receiving the in-person strategy did report fewer sunburns in the past year at posttest than those receiving the digital strategy (Table 3). By contrast, employees were more likely to report that they had received the GSS@W training for sun protection and other communication about sun protection at posttest in workplaces in the in-person rather than digital scalability strategy (Tables 4, 5). The employees were also more favorable toward the GSS@W training in in-person scalability workplaces at posttest. Employees’ reports of program implementation did not differ by wave (Supplemental Digital Content Table 2, http://links.lww.com/JOM/B735).

### Employee Sun Protection Behaviors

When posttested, employees indicated that they moderately frequently practiced sun protection at work (mean = 3.04 out of 5) (Table 2). In addition, more than half of employees experienced a sunburn in the past year (57.7%), with an average prevalence of 1.26 burns on the job in the past year (and 1.96 burns overall). There were no differences in employees’ sun protection at work by scalability strategy. Neither the main effect for treatment (Tables 3, 4) nor the interaction of treatment with wave on employees’ sun protection or sunburn prevalence at posttest were statistically significant (Supplemental Digital Content Table 2, http://links.lww.com/JOM/B735).

## DISCUSSION

### Principal Findings

Strategies for scalability of evidence-based interventions are an important but understudied aspect of dissemination and implementation science.^50^ This trial compared two scalability strategies for the GSS@W occupational sun protection intervention that differed in form of contact with employers (in-person versus digital), in this case regional districts within US state DOTs. In this trial, most managers reported at posttest that they implemented training and communicated with employees about sun protection through multiple workplace channels during the dissemination period (ie, since being pretested). Employees confirmed that they received this training and communication, and their reports of training and communication were higher in the in-person than digital scalability strategy. Employees also were more favorable toward training in the in-person than digital scalability strategy, too. The overall training rate was much higher than observed among a sample of state park workers.^91^ The more positive assessment of face-to-face on-site than digital training is consistent with another study in which employees desired hands-on training on prevention of occupational skin diseases, especially with visual content or a multimodel presentation.^92^ There may have been technological barriers to implementing digital training (eg, computers available only in central buildings rather than at the outdoor work areas) that reduced its use by managers in the digital scalability strategy districts.

In comparison to training and communication, managers may have implemented fewer actions related to protecting employees, such as providing personal protection equipment (PPE: sunscreen, clothing, hats, and eyewear), providing shade in the workplace, monitoring the UV Index, adjusting work schedules, conducting risk assessments, and encouraging employees to be screened for skin cancer. Provision of sunscreen at a worksite has increased the use of this personal protection item.^93^ When scaled-up, evidence-based interventions often are not implemented at levels seen in controlled evaluation studies.^50,52,53^ It is possible that managers adapted the GSS@W program to make it fit better with organizational procedures and technology, such as incorporating training and communication into routine training and safety communication channels and technologies (eg, learning management systems). Sun protection actions beyond training and communication may take more effort and have additional costs. While nearly three-quarters of managers said that there was a budget available for employee sun protection at posttest, we did not record its amount, and it may have been insufficient to cover a broad array of sun protection actions. Furthermore, employees reported that they had purchased very few items to assist themselves with sun protection. In addition, some sun protection equipment may not be easily and safely portable (eg, temporary shade canopies) in the far-flung DOT worksites.

The failure to affect employees’ sun protection occurred despite the greater implementation and training reported by employees in the in-person than digital scalability strategy. The findings are inconsistent with favorable outcomes from widescale implementation of occupational sun protection program in Denmark that included health education, personal dosimetry, and skin examinations.^43^ Reduced implementation and adaptations that lead to lower program fidelity can undermine a program’s effectiveness relative to that achieved in rigorous evaluation trials.^50,52,53^

### Impact of COVID-19 Restrictions

The results of this trial must be interpreted considering the substantial impacts on DOT workplaces, managers, and employees of the COVID-19 pandemic that interfered with the scalability strategies and implementation of GSS@W. Prior to the pandemic, intervention staff engaged in disseminating the GSS@W program to DOT districts in several states and it appeared that managers may have responded by implementing the GSS@W during this time (ie, in Wave 1). While many DOT employees were designated as essential workers during the initial pandemic period and were still at work, DOTs made several changes that made it impossible to deploy the in-person scalability strategy. Nearly all DOTs prohibited on-site visits by outside individuals, including the Sun Safety Coordinators, in some periods of Wave 2. We restarted the in-person visits when initial restrictions were relaxed but suspended them again during the surge of COVID-19 Omicron infections (for Waves 2 and 3). We resumed the in-person strategy after the Omicron surge; however, this inconsistent access to the workplaces undoubtedly weakened the in-person scalability strategy.

A second pandemic impact was that many DOTs suspended in-person gatherings of employees. This made GSS@W trainings difficult to implement on-site in district staff meetings because most meetings were canceled. While this was a barrier to the in-person scalability strategy, it also likely reduced training in the digital scalability strategy in workplaces that depended on training groups of employees and had limited technology to deploy the online GSS@W training. However, suspending in-person gatherings may have increased dependence on and familiarity with digital communication in the DOTs, reducing the functional difference between the in-person and digital scalability strategies.

A third impact of the pandemic was the psychological discordance between increased concern about masking, human interactions, and other restrictions intended to reduce the immediate risk of COVID-19 infection relative to taking actions on long-term risks associated with solar UV exposure. While Sun Safety Coordinators attempted to stay in contact with DOT management and motivate them to implement GSS@W, the added work tasks and challenges for managers, turnover in DOT staff, restrictions on on-site visits, and anxiety about COVID-19 infections anecdotally appeared to reduce managers’ commitment to GSS@W implementation. These pandemic disruptions illustrate that dissemination of evidence-based programs are subject to extraneous factors (the pandemic being an extreme case) that can and will dilute implementation and impact of a prevention program.

The COVID-19 pandemic response also weakened the trial design. We were told anecdotally that many managers and employees left the DOTs during the pandemic, retiring, leaving to care for family members, or separating due to personal outcomes of COVID-19 infections (eg, one DOT reported loss of approximately 750 employees in the first year of the pandemic). This workforce reduction possibly explained the high loss-to-follow-up rate among managers and lower number of planned employees posttested, which reduced statistical power. As a result, we altered our analyses to test just the posttest responses rather than adjust for pretest measures. In this approach, we assumed that randomization balanced managers, employees, and regional districts on job, demographic, and workplace characteristics and on existing sun safety policies and actions in the districts at baseline. This preserved the experimental nature of the trial design^90^ and was similar to our posttest-only analysis of employees in our effectiveness trial.^49^ However, it limited our conclusions about the success or failure of the scalability strategies and GSS@W to group differences in manager and employee reports after the dissemination period, rather than change in these reports from baseline.

Despite these pandemic interferences, we detected a difference in program implementation reported by employees at posttest by scalability strategy. This might suggest that the in-person scalability strategy’s improvement of GSS@W implementation was large and robust compared to the digital scalability strategy. However, the impact of the training and communication on employee sun protection behavior may have been reduced by employees being distracted by pandemic restrictions and personal precautions to avoid immediate exposure to the virus. Finally, the cost burden of pandemic response and restrictions was likely to have diverted resources from other workplace safety actions, such as sun safety, which may further explain why DOTs conducted training and communication rather than implement sun protection actions that involved providing personal protection equipment or altering work environments.

### Limitations

Beyond the deleterious effects of the pandemic response, there were other limitations to this trial. The study only examined scalability in a single public-sector transportation industry. Public employers may have few resources for new programs but more interest in program evaluation than private employers. However, the homogeneity of the DOT workplaces was an advantage for deploying the scalability strategy and improved the outcome analyses. The state DOT management structure commonly placed policy making at the state rather than district level. Thus, the number of policies was very small (*n* = 21) and could not be compared between the scalability strategies that were assigned to different districts within each state. Third, implementation and sun protection behaviors were self-reported. The implementation measure was based on one used in our effectiveness trial evaluating GSS@W, but self-reports can suffer from social desirability biases and memory errors, which may have been more likely when posttesting was delayed by pandemic restrictions. Unfortunately, our plans to inspect the worksites for GSS@W materials could not be performed during the pandemic. As noted, we did used sun protection behavior measures that have shown validity in past studies.

The trial had strengths. The sample was large. The state DOTs were located in all four US Census regions and included large and small states (based on geography and population) and states with diverse climates, latitudes, and, thus, levels of UV. DOTs served all areas of their states, so we reached rural as well as urban workers. Rural adults may practice sun protection less often than urban adults.^94^ The trial involved a randomized, prospective design, an experimental design preserved in the posttest-only analysis. The scalability strategies were selected to be typical of ways programs such as GSS@W can be distributed by public health departments and private vendors.

## CONCLUSIONS

To achieve the benefits of investments to produce effective evidence-based health interventions, it is essential to identify effective methods for distributing them widely to organizations and at-risk populations with sufficient implementation to benefit them. While relying on the latest computer and Internet technology to virtualize distribution holds promise, methods must be designed that achieve sufficient implementation of intervention activities if organizations adapt them in ways that reduce fidelity or effectiveness or when external factors interfere with intervention delivery.

## Supplementary Material

implementation_of_an_occupational_sun_safety_Table1

implementation_of_an_occupational_sun_safety_Table2

implementation_of_an_occupational_sun_safety_Figure2

implementation_of_an_occupational_sun_safety_Figure1

Supplemental digital contents are available for this article. Direct URL citation appears in the printed text and is provided in the HTML and PDF versions of this article on the journal’s Web site (www.joem.org).

## Figures and Tables

**TABLE 1.** Descriptive Statistics for Trial Design Features and Sample Demographics and Job Characteristics—*n* (%);Mean (SD)

	Managers (*n* = 255)	Employees (*n* = 1387)

Experimental design feature		
Dissemination strategy (treatment group)		
Digital	166 (65.1%)	862 (62.1%)
In-person	89 (35.9%)	525 (38.9%)
Study wave		
Wave 1 (pre-COVID)	117 (46.1%)	979 (70.6%)
Wave 2 (early COVID)	72 (28.2%)	240 (17.3%)
Wave 3 (late COVID)	66 (26.7%)	168 (12.1%)
Demographics		
Fitzpatrick skin type		
Always burn and is unable to tan	14 (5.6%)	86 (6.7%)
Usually burns but can tan if you work at it	54 (21.6%)	267 (20.6%)
Sometimes mildly burns and then tans easily	111 (44.4%)	507 (39.2%)
Rarely burns and tans easily	71 (28.4%)	433 (33.5%)
History of skin cancer		
No	219 (87.3%)	1188 (92.2%)
Yes	32 (12.7%)	100 (7.8%)
Gender		
Female	29 (11.5%)	167 (13.1%)
Male	224 (88.5%)	1109 (86.9%)
Race		
Non-White	20 (8.2%)	182 (16.7%)
White	223 (91.8%)	909 (83.3%)
Age (mean years, SD)	51.3 (8.19)	45.4 (12.04)
Job characteristics		
Number of years working for the DOT (mean [range = <1–40], SD)	18.78 (9.27)	
Directly supervise day-to-day activities of employees who work outdoors		
No	23 (23.3%)	
Yes	80 (77.7%)	
Hours worked outside per week (mean count [range = 0–40], SD)	17.37 (12.42)	23.45 (13.96)
Changes at the worksite related to COVID-19	189 (77.5%)	
Reduced number of employees	95 (41.0%)	
who work together	145 (60.7%)	
Reduced number of employees	194 (79.2%)	
who work on a shift	96 (41.6%)	
Closed indoor areas where employees gather (eg, before a shift; for a break or meal)	101 (43.7%)	
Prohibited employees from gathering together in groups of a certain large size	147 (61.3%)	
Postponed or eliminated certain job tasks	78 (34.1%)	
Postponed or eliminated safety	4.10 (2.52)	
and/or other trainings		
Changed to providing individual training online to employees rather than in groups in-person		
Postponed or eliminated large jobs that require large number of employees to work together		
Sum of Changes at Worksite Related to COVID-19 (mean count [range = 0–8], SD)		

**TABLE 2.** Descriptive Statistics for Implementation, Sun Protection, and Policy Awareness Outcomes—*n* (%); Mean (SD)

	Managers (*n* = 255)	Employees (*n* = 1387)

Sun safe workplaces program implementation		
Training and communication implementation score (mean = 2.68, SD = 1.39)		
0	28 (11.0%)	
1	27 (10.6%)	
2	32 (12.5%)	
3	60 (23.5%)	
4	108 (42.4%)	
Sun protection implementation score (mean = 1.66, SD = 1.33)		
0	51 (20.0%)	
1	80 (31.4%)	
2	61 (23.9%)	
3	36 (14.1%)	
4	17 (6.7%)	
5	10 (3.9%)	
Sum of innovation characteristics of implementing sun protection at work (mean ratings [range = 1–5], SD)	3.51 (0.56)	
Budget availability for sun protection		
No	158 (62.0%)	
Yes	97 (38.0%)	
Received GSS@W training		
No		649 (48.6%)
Yes		687 (51.4%)
Sum of sun protection information received at workplace (mean [range = 1–3], SD)		1.53 (1.22)
Favorability toward GSS@W training composite score (mean [range = 1–5], SD)		3.65 (0.54)
Personal sun protection behavior		
Personal sun protection behaviors composite score (mean frequency rating [range = 1–5], SD)	3.30 (0.63)	3.04 (0.73)
Any sunburn in past year		
No	138 (54.5%)	574 (42.3%)
Yes	115 (45.5%)	782 (57.7%)
Number of sunburns in past year (mean count, SD)	1.20 (2.12)	1.96 (2.83)
Number of sunburns in past year while working outside (mean count, SD)	0.87 (1.89)	1.26 (2.45)
Purchased sun protection items score (mean count [range = 0–2], SD)		0.70 (0.47)
Sun protection policy awareness		
Awareness of written sun protection policy		
No	178 (72.7%)	
Yes	67 (27.3%)	
Awareness of unwritten sun protection procedures		
No	151 (60.9%)	
Yes	97 (39.1%)	

**TABLE 3.** Implementation, Sun Protection, and Policy Outcomes for Managers by Dissemination Strategy and Wave—*n/n* (%); Mean (SD)

	Wave 1 (Pre-COVID) – Managers		Wave 2 (Early COVID) – Managers		Wave 3 (Late COVID) – Managers	
			
Outcome	Digital (*n* = 74)	In-Person (*n* = 43)	Digital (*n* = 48)	In-Person (*n* = 24)	Digital (*n* = 44)	In-Person (*n* = 22)

Sun safe workplaces program implementation						
Training and communication implementation score^a^	2.47 (1.38)	2.95 (1.40)	2.83 (1.40)	2.75 (1.39)	2.86 (1.37)	2.95 (1.33)
Sun protection implementation score^b^	1.31 (0.98)	1.51 (1.16)	1.58 (1.32)	1.88 (1.30)	2.32 (1.62)	1.95 (1.68)
Innovation characteristics of implementing sun protection at work^c^	3.50 (0.50)	3.68 (0.60)	3.42 (0.61)	3.33 (0.69)	3.63 (0.58)	3.73 (0.67)
Budget available for employee sun protection	23/74 (31.1%)	17/43 (39.5%)	17/48 (35.4%)	11/24 (45.8%)	20/44 (45.5%)	9/22 (40.9%)
Personal sun protection behavior						
Personal sun protection behaviors scale score^d^	3.23 (0.55)	3.26 (0.69)	3.27 (0.62)	3.25 (0.69)	3.38 (0.64)	3.61 (0.69)
Number of sunburns in past year	1.99 (2.69)	0.95 (1.41)	0.55 (0.97)	0.83 (1.27)	1.14 (2.28)	0.90 (2.62)
Number of sunburns in past year while working outside	1.58 (2.62)	0.69 (1.17)	0.30 (0.79)	0.61 (0.99)	0.79 (2.23)	0.43 (0.87)
Any sunburn in past year	45/73 (61.6%)	20/43 (46.5%)	15/48 (31.3%)	9/24 (37.5%)	19/43 (44.2%)	7/22 (31.8%)
Workplace sun protection policy						
Knowledge of workplace sun protection policy score^6^	1.47 (2.47)	1.84 (3.03)	1.34 (2.49)	1.04 (2.39)	1.98 (3.42)	2.65 (3.62)

a Sum of four occupational sun protection training and communication items (range = 0–4).

b Sum of six occupational sun protection action items (range = 0–6).

c Average of six Likert-type items (range = 1–5).

d Average of nine frequency items (range = 1–5).

**TABLE 4.** Main Effect Test Comparing Dissemination Strategy (In-Person vs Digital) on Program Implementation and Occupational Sun Protection Outcomes for Managers and Employees (Regression Coefficient, *P* [Two-Tailed])

Outcome	Managers (*n* = 255)	Employees (*n* = 1387)

Sun safe workplaces program implementation		
Training and communication implementation score^a^	0.050, *P* = 0.50	
Sun protection implementation score^a^	0.017, *P* = 0.88	
Innovation characteristics of implementing sun protection at work^b^	0.055, *P* = 0.56	
Budget availability for sun protection^c^	0.175, *P* = 0.53	
Received GSS@W training^c^		0.381, *P* = 0.021*
Favorability toward GSS@W training^3^		0.087, *P* = 0.022*
Sun protection information received at workplace^a^		0.112, *P* = 0.049*
Personal sun protection behavior		
Personal sun protection behaviors scale score^b^	0.122, *P* = 0.13	0.055, *P* = 0.25
Number of sunburns in past year^a^	−0.105, *P* = 0.65	−0.125, *P* = 0.20
Number of sunburns in past year while working outside^a^	0.100, *P* = 0.72	−0.147, *P* = 0.29
Any sunburn in past year^c^	−0.180, *P* = 0.59	0.124, *P* = 0.46
Purchased sun protection items score^b^		0.031, *P* = 0.35
Workplace sun protection policy		
District sun protection policies^a^	−0.051, *P* = 0.87	

* *P* < 0.05 (two-tailed).

Note: Treatment effect represents the effect averaged across wave and adjusting for the control variables (hours worked outside, age, skin type, gender, and race/ethnicity).

a Log odds coefficient from negative binomial regression model.

b Linear regression coefficient.

c Log odds coefficient from logistic regression.

**TABLE 5.** Implementation, Sun Protection, and Policy Outcomes for Employees by Dissemination Strategy and Wave—*n/n* (%); Mean (SD)

	Wave 1 (Pre-COVID) – Employees		Wave 2 (Early COVID) – Employees		Wave 3 (Late COVID) – Employees	
			
Outcome	Digital (*n* = 640)	In-Person (*n* = 339)	Digital (*n* = 125)	In-Person (*n* = 115)	Digital (*n* = 97)	In-Person (*n* = 71)

Sun safe workplaces program implementation						
Received go sun smart at work training	245/607 (40.4%)	183/324 (56.5%)	73/124 (58.9%)	69/113 (61.1%)	63/97 (64.9%)	54/71 (76.1%)
Favorability toward go sun smart at work training^a^	3.55 (0.53)	3.65 (0.52)	3.58 (0.50)	3.56 (0.56)	3.81 (0.49)	3.99 (0.55)
Sun protection information received at workplace^b^	1.37 (1.20)	1.44 (1.23)	1.63 (1.24)	2.02 (1.13)	1.77 (1.18)	2.07 (1.07)
Personal sun protection behavior						
Personal sun protection behaviors scale score^c^	2.94 (0.71)	2.94 (0.75)	3.24 (0.77)	3.42 (0.73)	3.19 (0.59)	3.16 (0.64)
Number of sunburns in past year	2.2 (3.1)	1.8 (2.6)	2.2 (3.3)	1.8 (2.1)	1.45 (2.21)	0.99 (1.55)
Number of sunburns in past year while working outside	1.49 (2.83)	1.19 (2.14)	1.49 (2.69)	1.02 (1.61)	0.63 (1.70)	0.36 (0.91)
Any sunburn in past year	380/623 (61.0%)	185/327 (56.6%)	68/124 (54.8%)	78/115 (67.8%)	45/96 (46.9%)	26/71 (36.6%)
Purchased sun protection items^d^	0.66 (0.42)	0.64 (0.50)	0.72 (0.46)	0.88 (0.53)	0.79 (0.50)	0.78 (0.49)

a Average of five Likert-type items (range = 1–5).

b Sum of three types of communication (range = 0–3).

c Average of nine sun protection behavior frequency items (range = 1–5).

d Sum of four personal sun protection items (range = 0–4).

## Data Availability

The data set generated and analyzed are available in the Interuniversity Consortium for Political and Social Research repository (https://www.icpsr.umich.edu).

## LEARNING OUTCOMES

- To describe two methods for disseminating an occupational skin cancer prevention program nationwide, relying on in-person and digital contact, to state Departments of Transportation.
- To summarize increases in measures of implementation of occupational sun protection by managers supervising outdoor workers and employees and differences associated with the two scalability strategies.
- To recognize the importance of effective scalability strategies for achieving the benefits of evidence-based cancer prevention programs in community-wide dissemination.
