# Supplementary material for: Implementation of an Occupational Sun Safety Intervention: A Comparison of Two Scalability Strategies
Source: J Occup Environ Med. Author manuscript; Available in PMC 2026 Jul 16. (PMC13375161; doi:10.1097/JOM.0000000000003248)
Supplement: implementation_of_an_occupational_sun_safety_Table1 [file NIHMS2166091-supplement-implementation_of_an_occupational_sun_safety_Table1.pdf]

Supplemental Digital Content Table 1: Full models estimating treatment effect on managers' program implementation and sun protection behavior outcomes (regression coefficients,[1] p [two-tailed])

Managers (N = 255)			
**Effect**	**Training and Communication Implementation Score**	**Sun Protection Implementation Score**	**Innovation Characteristics of Implementing Sun Protection at Work**
**Treatment**	0.050, p=0.50	0.017, p=0.88	0.055, p=0.56
**Wave 1**	−0.028, p=0.66	−0.146, p=0.11	0.099, p=0.23
**Wave 2**	0.066, p=0.32	0.029, p=0.76	−0.121, p=0.14
**Treatment X Wave 1**	0.085, p=0.40	0.028, p=0.85	0.027, p=0.82
**Treatment X Wave 2**	−0.090, p=0.41	0.149, p=0.43	−0.126, p=0.37
**Intercept**	0.975, p<0.001	0.459, p<0.001	3.503, p<0.001

[1]Regression coefficients in natural log scale from a negative binomial model.

	**Managers (N = 255)**		
**Effect**	**Budget Availability for Sun Protection**	**Personal Sun Protection Behaviors Scale Score**	**Number of Sunburns in Past Year**
**Treatment**	0.175, p=0.53	0.122, p=0.13	−0.105, p=0.65
**Wave 1**	0.340, p=0.25	0.001, p=0.99	0.614, p=0.002*
**Wave 2**	−0.281, p=0.25	−0.055, p=0.47	−0.393, p=0.040*

**Treatment X Wave 1**	0.009, p=0.98	−0.114, p=0.26	−0.705, p=0.011*
**Treatment X Wave 2**	0.264, p=0.51	−0.052, p=0.64	0.444, p=0.16
**Intercept**	−0.727, p<0.001	3.270, p<0.001	−0.188, p=0.16

**Managers (N = 255)**			
**Effect**	**Number of Sunburns in Past Year While Working Outside**	**Any Sunburn in Past Year**	**District Sun Protection Policies**
**Treatment**	0.100, p=0.72	−0.180, p=0.59	−0.051, p=0.87
**Wave 1**	0.821, p=0.001*	0.832, p=0.014*	−0.022, p=0.94
**Wave 2**	−0.603, p=0.029*	−0.524, p=0.06	−0.112, p=0.66
**Treatment X Wave 1**	−0.868, p=0.009*	−0.711, p=0.10	−0.039, p=0.92
**Treatment X Wave 2**	0.733, p=0.08	0.608, p=0.20	0.144, p=0.73
**Intercept**	−0.726, p<0.001	−0.299, p=0.09	0.200, p=0.22

*p<0.05 (two-tailed)
