# Supplementary material for: Implementation of an Occupational Sun Safety Intervention: A Comparison of Two Scalability Strategies
Source: J Occup Environ Med. Author manuscript; Available in PMC 2026 Jul 16. (PMC13375161; doi:10.1097/JOM.0000000000003248)
Supplement: implementation_of_an_occupational_sun_safety_Table2 [file NIHMS2166091-supplement-implementation_of_an_occupational_sun_safety_Table2.pdf]

Supplemental Digital Content Table 2: Full models estimating treatment effect on employees' program implementation and sun protection behavior outcomes (regression coefficients,[1] p [two-tailed])

Employees (N = 1387)			
**Effect**	**Received GSS@W Training**	**Favorability Toward GSS@W Training**	**Sun Protection Information Received at Workplace**
**Treatment**	0.381, p=0.021*	0.087, p=0.022*	0.112, p=0.049*
**Wave 1**	−0.393, p=0.06	−0.087, p=0.025*	−0.079, p=0.15
**Wave 2**	0.064, p=0.81	−0.060, p=0.241*	0.000, p=0.99
**Treatment X Wave 1**	0.192, p=0.38	0.019, p=0.71	−0.095, p=0.19
**Treatment X Wave 2**	−0.285, p=0.33	−0.110, p=0.09	0.107, p=0.28
**Intercept**	0.106, p=0.49	3.644, p<0.001	0.424, p<0.001

[1]Regression coefficients in natural log scale from a negative binomial model.

	**Employees (N = 1387)**		
**Effect**	**Personal Sun Protection Behaviors Scale Score**	**Number of Sunburns in Past Year**	**Number of Sunburns in Past Year While Working Outside**
**Treatment**	0.055, p=0.25	−0.125, p=0.20	−0.147, p=0.28
**Wave 1**	−0.179, p=0.001*	0.018, p=0.85	0.098, p=0.34
**Wave 2**	0.115, p=0.07	0.183, p=0.12	0.351, p=0.014*
**Treatment X Wave 1**	−0.022, p=0.74	0.052, p=0.62	0.098, p=0.52
**Treatment X Wave 2**	0.105, p=0.17	−0.099, p=0.45	−0.240, p=0.19

**Intercept**	3.115, p<0.001	0.483, p<0.001	−0.177, p=0.033

Employees (N = 1387)		
**Effect**	**Any Sunburn in Past Year**	**Purchased Sun Protection Items**
**Treatment**	0.124, p=0.46	0.031, p=0.35
**Wave 1**	0.132, p=0.39	−0.085, p=0.001*
**Wave 2**	0.087, p=0.67	−0.015, p=0.63
**Treatment X Wave 1**	−0.212, p=0.24	−0.066, p=0.10
**Treatment X Wave 2**	0.408, p=0.11	0.121, p=0.05
**Intercept**	0.323, p=0.011	0.745, p<0.001

*p<0.05 (two-tailed)
