# Supplementary figures and images for: Implementation of an Occupational Sun Safety Intervention: A Comparison of Two Scalability Strategies
Source: J Occup Environ Med. Author manuscript; Available in PMC 2026 Jul 16. (PMC13375161; doi:10.1097/JOM.0000000000003248)

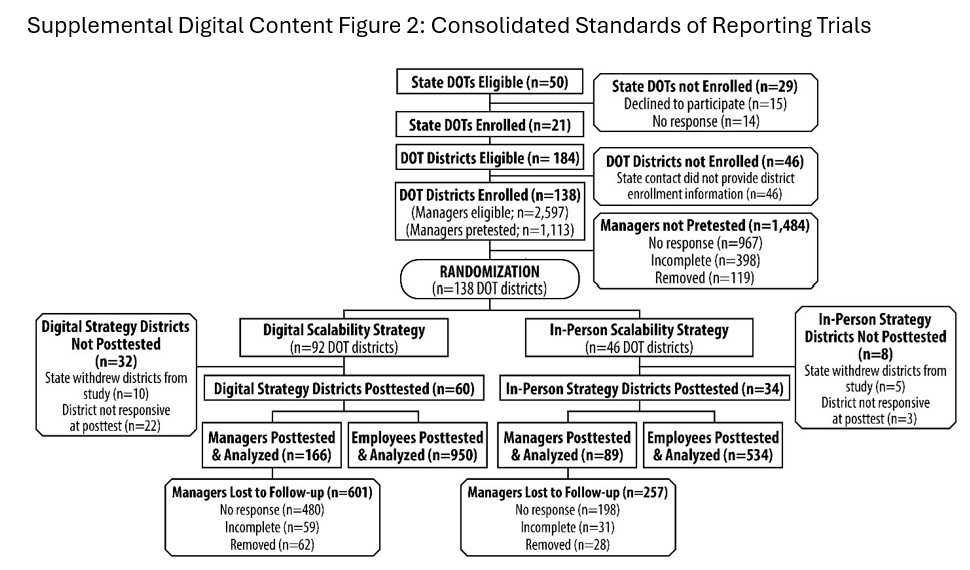

Supplement: implementation_of_an_occupational_sun_safety_Figure2 [file NIHMS2166091-supplement-implementation_of_an_occupational_sun_safety_Figure2.jpeg]

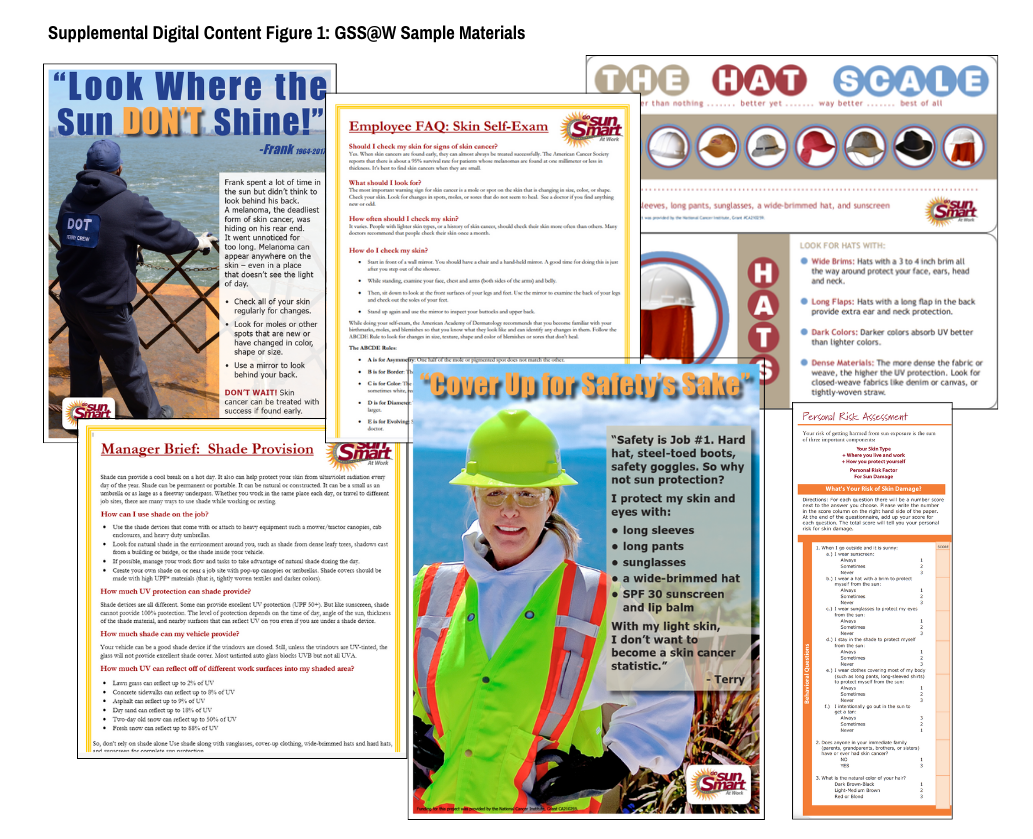

Supplement: implementation_of_an_occupational_sun_safety_Figure1 [file NIHMS2166091-supplement-implementation_of_an_occupational_sun_safety_Figure1.jpeg]
